# Resveratrol enhances the antiliver cancer effect of cisplatin by targeting the cell membrane protein PLA2

**DOI:** 10.3389/fonc.2024.1453164

**Published:** 2024-09-24

**Authors:** Yu Gao, Zhanyi Yang, Akhilesh Kumar Bajpai, Wenben Wang, Liyuan Zhang, Zhenhong Xia

**Affiliations:** ^1^ Department of Pharmacy, Binzhou Medical University, Yantai, China; ^2^ Department of Genetics, Genomics and Informatics, University of Tennessee Health Science Center, Memphis, TN, United States; ^3^ Key Laboratory of Ion Beam Bioengineering, Hefei Institute of Physical Sciences, Chinese Academy of Sciences, Hefei, China

**Keywords:** combination therapy, resveratrol, cisplatin, anti-liver cancer mechanism, cell membrane, PLA2

## Abstract

**Background:**

In this study, we aimed to explore the mechanism by which resveratrol promotes cisplatin-induced death of HepG2 cells and to provide a potential strategy for resveratrol in the treatment of cancer.

**Methods:**

HepG2 cells were exposed to a range of drug concentrations for 24 h: resveratrol (2.5 μg/mL [10.95 μM], 5 μg/mL [21.91 μM], 10 μg/mL [43.81 μM], 20 μg/mL [87.62 μM], 40 μg/mL [175.25 μM], and 80 μg/mL [350.50 μM]), cisplatin (0.625 μg/mL [2.08 μM], 1.25 μg/mL [4.17 μM], 2.5 μg/mL [8.33 μM], 4.5 μg/mL [15.00 μM], and 10 μg/mL [33.33 μM]), 24 μg/mL (105.15 μM) resveratrol + 9 μg/mL (30.00 μM) cisplatin, and 12 μg/mL (52.57 μM) resveratrol + 4.5 μg/mL (15.00 μM) cisplatin. The interaction of two drugs was evaluated by coefficient of drug interaction (CDI), which was based on the Pharmacological Additivity model. The MTT 3-(4,5-dimethylthiazol-2-yl)-2,5-diphenyltetrazolium bromide assay was used to detect the effect of different concentrations of drugs on cell viability, while transcriptome sequencing was used to identify pathways associated with higher gene enrichment. Synchrotron radiation FTIR microspectroscopy experiments and data analysis were conducted to obtain detailed spectral information. The second-derivative spectra were calculated using the Savitzky–Golay algorithm. Single-cell infrared spectral absorption matrices were constructed to analyze the spectral characteristics of individual cells. The Euclidean distance between cells was calculated to assess their spectral similarity. The cell-to-cell Euclidean distance was computed to evaluate the spatial relationships between cells. The target protein of resveratrol was verified by performing a Western blot analysis.

**Results:**

After 24 h of treatment with resveratrol, HepG2 cell growth was inhibited in a dose-dependent manner. Resveratrol promotes cisplatin-induced HepG2 cell death through membrane-related pathways. It also significantly changes the membrane components of HepG2 cells. Additionally, resveratrol changes the morphology of the HepG2 cell membrane by decreasing the expression of PLA2G2.

**Conclusion:**

Resveratrol changes the morphology of the HepG2 cell membrane by decreasing the expression of PLA2G2 and promotes cisplatin-induced HepG2 cell death. The combination of cisplatin and resveratrol can play a synergistic therapeutic effect on HepG2 cells.

## Introduction

Hepatocellular carcinoma (HCC) is the fourth leading cause of cancer-related mortality worldwide and a major cause of death in cirrhosis. The prognosis for HCC is poor, with mortality rates approaching incidence rates globally (1). According to the Barcelona Clinic Cancer (BCLC) staging system (2–4), in principle, patients with early-stage HCC tumors are generally preferred candidates for resection, transplantation, and local ablation. In contrast, patients at intermediate stages are typically considered first candidates for transcatheter arterial chemoembolization (TACE), while those with advanced disease are usually the first to receive systemic therapies (5). Chemotherapy is one of the most common methods of systemic therapies; however, advanced HCC is often chemo-resistant, which limits the available therapeutic options for patients (6). Cisplatin-based chemotherapy is a common treatment for HCC (7), but cisplatin resistance is the main obstacle in treating HCC. Li et al. suggested that circMRPS35 plays an important role in cisplatin resistance (8), while Tang et al. proposed that HepG2/DDP cell-derived exosomes confer cisplatin resistance to HepG2 and other HCC cell lines (9). Ding et al. showed osteopontin (OPN) enhances chemoresistance of cisplatin in HCC cells by activating the PI3K/AKT signaling pathway (10). There is an urgent clinical need to find new strategies for overcoming cisplatin (CDDP) resistance and improving sequential therapy.

Due to the increasing acceptance of complementary and alternative treatment systems, natural and nutritional medicines are becoming popular as effective methods for cancer treatment (11, 12). Therefore, finding a suitable combination of Chinese and Western medicine is of great clinical significance for cancer prevention and treatment. Resveratrol (3,4′,5-trihydroxy-trans-stilbene [Res]), a polyphenolic natural compound abundantly found in plants, foods, and red wine, has received a lot of attention due to its cancer prevention and anticancer properties (13, 14). In addition, as a multitarget drug, resveratrol is well-suited for use in combination with other drugs (15), and there have been many reports on the combination of resveratrol and cisplatin. For example, resveratrol can overcome cisplatin resistance and prevent metastasis in oral squamous cell carcinoma (OSCC) cells (16); it enhances cisplatin toxicity in human hepatoma cells; it reduces cisplatin-induced ovarian damage (17); and it can antagonize cisplatin-induced ototoxicity (18). Combination therapy of cisplatin and resveratrol induced cellular aging in gastric cancer cells (19), but how resveratrol enhances the effect of cisplatin remains unclear. Phospholipase A2 enzymes (PLA2s) hydrolyze the sn-2 acyl bond of glycerophospholipids (GPLs) to release lysophospholipids (LPLs) and free fatty acids, mostly polyunsaturated fatty acids (PUFAs) (20). sn-2 LPLs can be converted into lysophosphatidic acids (LPAs) by lysophospholipase D (LPLD). LPAs are important bioactive lipids that are widely involved in various physiological processes, including cell proliferation, survival, apoptosis, cytoskeletal construction, inflammation, and cancer (21). These bioactive eicosanoid molecules widely participate in the processes of inflammation and carcinogenesis. The PLA2 superfamily comprises at least six big families of isoenzymes, classified based on their structure, location, substrate specificity, and physiologic roles, which are secreted PLA2 (sPLA2) (22), cytosolic PLA2 (cPLA2) (23), Ca^2+^-independent PLA2 (iPLA2) (24–26), lipoprotein-associated PLA2 (LpPLA2), lysosomal PLA2 (LPLA2), and adipose-tissue-specific PLA2 (AdPLA2), respectively. PLA2G2 is a secreted protein. In general, sPLA2 plays a protumorigenic role in breast, lung, prostate, ovarian, and esophageal cancers, while having an antitumorigenic role in gastric and intestinal cancers. cPLA2 is an important enzyme in AA metabolism, and although cPLA2α, cPLA2β, and cPLA2γ are all present in human cells, only cPLA2α plays a crucial role in the pathophysiology of inflammation-related diseases and cancer through the AA cascade pathway. Several *in vitro* studies have demonstrated that immortal cell lines (such as the U937 human histiocytic lymphoma cell line and HT-29 and DLD-1 colon cancer cell lines) express high levels of iPLA2β, and that iPLA2β inhibition induces apoptosis in cancer cells. Previous studies have shown that resveratrol can protect cell membranes from PLA2 damage (21, 27–30). We are interested in whether resveratrol enhances the antiliver cancer effect by targeting the PLA2G2.

Evaluating interactions between bioactive agents has become an important topic in many projects. The interactions between multiple drugs are usually divided into synergistic or antagonistic effects, and determining drug interactions is based on the null hypothesis of “no interaction”. According to the different null hypotheses, models for assessing interactions include Bliss’s (1939) statistical independent model, Gaddum’s (1940) Pharmacological Independence model, and Loewe’s (1928) Pharmacological Additivity model. Although each model has its own logical foundation, the fundamental assumptions behind these three models differ (31–33). The major differences between the three reference models come from their underlying assumptions. For example, the Bliss independence model assumed that drugs do not interact with each other and elicit their responses independently, whereas the Loewe additivity model assumes that drugs have similar modes of action on the same pathway. In fact, none of these models apply universally to all cases of drug combinations. As a result, the selection of model has become a personal preference (32).

The development of synchrotron radiation (SR)-based infrared spectroscopy has allowed for spatially resolved chemical mapping and molecular characterization of individual cells (34–37). Since the infrared spectra contain a lot of information about fatty acids, proteins, and nucleic acid–carbohydrates, Fourier transform infrared (FTIR) spectroscopy enables the detection of cellular composition, molecular structure, and biochemical changes in response to stimuli (38–41). As spectral diversity reflects relatively small biochemical changes in cells, single-cell FTIR can, in principle, be used to identify cell-to-cell variability that is lost in studies of average cell spectra.

In this study, we first used the Pharmacological Additivity model, Statistical Independence model, and Pharmacological Independence model to evaluate the effect of combination therapy with resveratrol and cisplatin. The MTT assay was used to detect the effect of different concentrations of drugs on cell viability, wgile transcriptome sequencing was used to identify pathways associated with higher gene enrichment. Synchrotron radiation FTIR microspectroscopy experiment and data analysis were conducted to obtain detailed spectral information. Savitzky–Golay algorithm was used to calculate the second-derivative spectra. The absorption matrix of the single-cell infrared spectrum was constructed to analyze the spectral characteristics of individual cells. The Euclidean distance between cells was calculated to assess their spectral similarity. The target protein of resveratrol was verified by performing a Western blot analysis. Exploring the mechanism by which resveratrol promotes cisplatin-induced death of HepG2 cells could provide a potential strategy for using resveratrol in the treatment of cancer.

## Materials and methods

### Cell culture

The human hepatoma cell line HepG2 was obtained from the Type Culture Collection of the Chinese Academy of Sciences (Shanghai, China) and cultured in DMEM supplemented containing 10% FBS, 100 units/mL of penicillin, and 100 µg/mL of streptomycin. The cells were incubated at 37°C in an atmosphere of 5% CO_2_. Tests for mycoplasma contamination were negative.

### Reagents and antibodies

3-(4,5-Dimethyl-2-thiazolyl)-2,5-diphnyl-2H-tetrazolium bromide (MTT) was purchased from Sigma (St. Louis, MO, USA). Anti-PLA2G2A antibodies were obtained from Amyjet Scientific (Wuhan, China), and anti-beta antibodies and the secondary antibody were purchased from abcam (Cambridge, UK). Resveratrol and cisplatin were also provided by Sigma (St. Louis, MO, USA). In the experiments, resveratrol was dissolved in dimethylsulfoxide (DMSO) at a storage solution concentration of 50 mg/mL and diluted to the desired concentrations with cell culture medium before use. Metformin was dissolved in deionized water at a storage solution concentration of 0.5 mg/mL and diluted to the desired concentration before use.

### Cell viability and IC_50_ assay

An MTT assay was used to evaluate the viability of HepG2 cells. Cells (6 × 10^3^ cells/well) were seeded in 96-well plates and incubated at 37°C for 24 h. Next, cells were exposed to resveratrol (2.5 μg/mL [10.95 μM], 5 μg/mL [21.91 μM], 10 μg/mL [43.81 μM], 20 μg/mL [87.62 μM], 40 μg/mL [175.25 μM], and 80 μg/mL [350.50 μM]), cisplatin (0.625 μg/mL [2.08 μM], 1.25 μg/mL [4.17 μM], 2.5 μg/mL [8.33 μM], 4.5 μg/mL [15.00 μM], and 10 μg/mL [33.33 μM]), 24 μg/mL (105.15 μM) resveratrol + 9 μg/mL (30.00 μM) cisplatin, and 12 μg/mL (52.57 μM) resveratrol + 4.5 μg/mL (15.00 μM) cisplatin. A total of 20 μL of MTT solution (5 mg/mL [12.7 μM]) was added to each well and incubated for 4 h at 37°C. Next, 150 μL of DMSO was added to each well to dissolve the formazan. Absorbance was recorded at 490 nm using a microtiter plate reader (BioTek, Vermont, USA). Each experiment was repeated three times. The IC_50_ values were calculated using GraphPad Prism software (GraphPad Inc., La Jolla, CA, USA).

### Drug interaction assay

Cells (6 × 10^3^ cells/well) were seeded in 96-well plates and incubated at 37°C for 24 h. They were then exposed to different concentrations of resveratrol and cisplatin for an additional 24 h. The treatments included resveratrol (24 μg/mL [105.15 μM] and 12 μg/mL [52.57 μM]), cisplatin (9 μg/mL [30.00 μM] and 4.5 μg/mL [15.00 μM]), 24 μg/mL (105.15 μM) resveratrol + 9 μg/mL (10.95 μM) cisplatin and 12 μg/mL (52.57 μM) resveratrol + 4.5 μg/mL cisplatin (15.00 μM). A total of 20 μL of MTT solution (5 mg/mL [12.7 μM]) was added to each well and incubated for 4 h at 37°C. Next, 150 μL of DMSO was added to each well to dissolve the formazan. Absorbance was recorded at 490 nm using a microtiter plate reader (BioTek, USA). Each experiment was repeated three times. The interaction between two drugs was evaluated using the coefficient of drug interaction (CDI), which is based on the Pharmacological Additivity model (42, 43), Statistical Independence model (44), and Pharmacological Independence model, respectively. CDI was calculated using the formula: CDI = P (a + b)/(Pa × Pb), where P (a + b) represents the cell viability after treatment with both drugs A and B, while Pa and Pb represent cell viability after treatment with a single compound alone. CDI < 1 represents the synergy of A and B, CDI = 1 represents the additivity of A and B, and CDI > 1 represents the antagonism of A and B. According to the Statistical Independence model, if the probabilities of death are statistically independent, then P (a + b) = 1 − (1 − Pa) × (1 − Pb), which means there is no interaction between the two drugs. If P(a + b) > 1 − (1 − Pa) × (1 − Pb), it indicates a synergistic effect between these two drugs. Conversely, if P(a + b) < 1 − (1 − Pa) × (1 − Pb), these two drugs exhibit antagonism.

### RNA sequencing analysis

HepG2 cells in 60 mm cell culture dishes were treated with different concentrations of drugs. The HepG2 cells were treated with 24 μg/mL (105.15 μM) resveratrol (R group), 9 μg/mL (30.00 μM) cisplatin (C group), and a combination of 24 μg/mL (105.15 μM) resveratrol + 9 μg/mL (30.00 μM) cisplatin (CR group) for 24 h. Three biological replicates were performed for each group. After treatment, cells were collected using TRIzol reagent. RNA sequencing (RNA-seq) analysis was performed at YuanXin Biotech (Shanghai, China).

### Sample preparation for infrared microspectroscopy

HepG2 cells were exposed to a range of drug concentrations for 24 h: resveratrol (24 μg/mL [105.15 μM]), cisplatin (9 μg/mL [30.00 μM]), and a combination of 24 μg/mL (105.15 μM) resveratrol + 9 μg/mL (30.00 μM) cisplatin. After treatment, the HepG2 cells were washed three times with PBS, then detached from the cell culture dishes using cell scrapers and transferred into centrifuge tubes. The fixed cells were washed three times and resuspended in 20 μL of ultrapure water. Next, 3 μL of the cell suspension was dropped on a barium fluoride (BaF2) window and deposited under room temperature for at least 30 min until completely dried. Single cells were expected to disperse on the window.

### Synchrotron radiation FTIR microspectroscopy experiments and data analysis

HepG2 cells were exposed to a range of drug concentrations for 24 h: resveratrol (24 μg/mL [105.15 μM]), cisplatin (9 μg/mL [30.00 μM]), and a combination of 24 μg/mL (105.15 μM) resveratrol + 9 μg/mL (30.00 μM) cisplatin. After treatment, SR-FTIR was carried out at the beamline BL01B of Shanghai Synchrotron Radiation Facility (SSRF), which is equipped with a Nicolet 6700 Fourier transform infrared spectrometer, a continuum infrared microscope, and a ×32 infrared objective. To ensure that the collected spectra of single cells had comparable signal intensities and signal/noise ratios, a consistent aperture size of 20 µm × 20 μm was used throughout the experiment. All the spectra were obtained within the mid-infrared region of 4,000−650 cm^−1^ with a resolution of 4 cm^−1^ and with 256 coadded scans for each spectrum. The original spectra were smoothed (9-point) and underwent linear automatic baseline correction. The second-derivative spectra were calculated using the Savitzky–Golay algorithm. All data collection and preprocessing procedures were operated on OMNIC (Thermo Fisher Scientific, Waltham, USA). Infrared spectral Mie scattering was corrected on MatLab R2014a using Resonant Mie Scattering EMSC (RMieS-EMSC) correction (45). Principle component analysis (PCA) was also carried out on MatLab.

### Construction of single-cell infrared spectral absorption matrices

Single-cell infrared spectra obtained at different incubation time points were randomly selected to construct the spectral matrices. A total of 14 wavenumber values corresponding to the minimum points in the second-derivative spectra (which correspond to absorption peak positions in the original spectra) were extracted each cell. Thus, a spectral matrix with a 14-dimensional vector was obtained using the extracted values from the infrared spectra of each cell.

### Calculation of the cell-to-cell Euclidean distance

The cell-to-cell Euclidean distances were evaluated using the following formula:


$$D_{\text{Eu}}{\left(p,q\right)}^{=}\sqrt{\sum_{i=1}^{n}{\left(pi-qi\right)}^{2},}$$

where *D*_Eu_(*p*, *q*) is the cell-to-cell Euclidean distance, and *p* and *q* are *n*-dimensional vectors of two different cells. In the case when the cell number is *k*, a total of *k^2^* cell-to-cell distances are calculated to construct the heatmaps. Among these, the *C_k_^2^* distance values were used to analyze the distribution in a histogram. The whole spectrum and the subregional spectra were used to calculate the cell-to-cell distances. When the subregional spectra were used, a four-dimensional vector for the fatty acid region (peak 1 to peak 4), a seven-dimensional vector for the protein region (peaks 5 to 11), and a three-dimensional vector for the nucleic acid-carbohydrate region (peak 12 to peak 14) were obtained. The value of the Euclidean distance is negatively correlated with the cell-to-cell similarity.

### Western blot analysis

Cells were exposed to a range of drug concentrations, including 24 h resveratrol (24 μg/mL [105.15 μM] and 12 μg/mL [52.57 μM]), cisplatin (9 μg/mL [30.00 μM] and 4.5 μg/mL [15.00 μM]), and combinations of 24 μg/mL (105.15 μM) resveratrol + 9 μg/mL (30.00 μM) cisplatin and 12 μg/mL (52.57 μM) resveratrol + 4.5 μg/mL (15.00 μM) cisplatin. A 20-μL MTT solution (5 mg/mL [12.7 mM]) was used to assess HepG2 cells exposed to a range of drug concentrations. The cells were treated with resveratrol (24 μg/mL [105.15 μM]), cisplatin (9 μg/mL [30.00 μM]), or a combination of 12 μg/mL (52.57 μM) resveratrol + 4.5 μg/mL (15.00 μM) cisplatin for durations of 3, 6, 12, and 24 h. After treatment, HepG2 cells were harvested and lysed using lysis buffer Beyotime (Shanghai, CHN) at 4°C. The lysates were centrifuged at 15,000 rpm for 15 min at 4°C. The supernatants were quantified using the BCA Assay Kit (P0009) (Beyotime, Shanghai, China). The same amount of protein samples were subjected to 10% SDS-PAGE gel and transferred to the PVDF membranes. The membranes were sealed with 5% skimmed milk at room temperature for 2 h and then incubated overnight with the corresponding primary antibodies at 4°C. Next, the membranes were incubated with horseradish peroxidase-conjugated secondary antibodies at room temperature for 2 h. Images were generated using the Western Blot Detection System (ImageQuant LAS 500, USA). The relative expression of proteins was standardized with β-actin, which is used as an internal reference.

### Statistical analysis

All experiments were repeated independently three times unless specifically indicated otherwise. The data were analyzed using GraphPad Prism 7 software and shown as mean ± SD. The Student’s *t*-test was used to determine the levels of significance between comparison samples. *p* > 0.05 was not significant (ns). Significance levels are indicated as follows: ^*^ *p* < 0.05, ^**^ *p* < 0.01, and ^***^ *p* < 0.001.

## Result

### Resveratrol promotes cisplatin-induced HepG2 cell death

**Figure 1:**
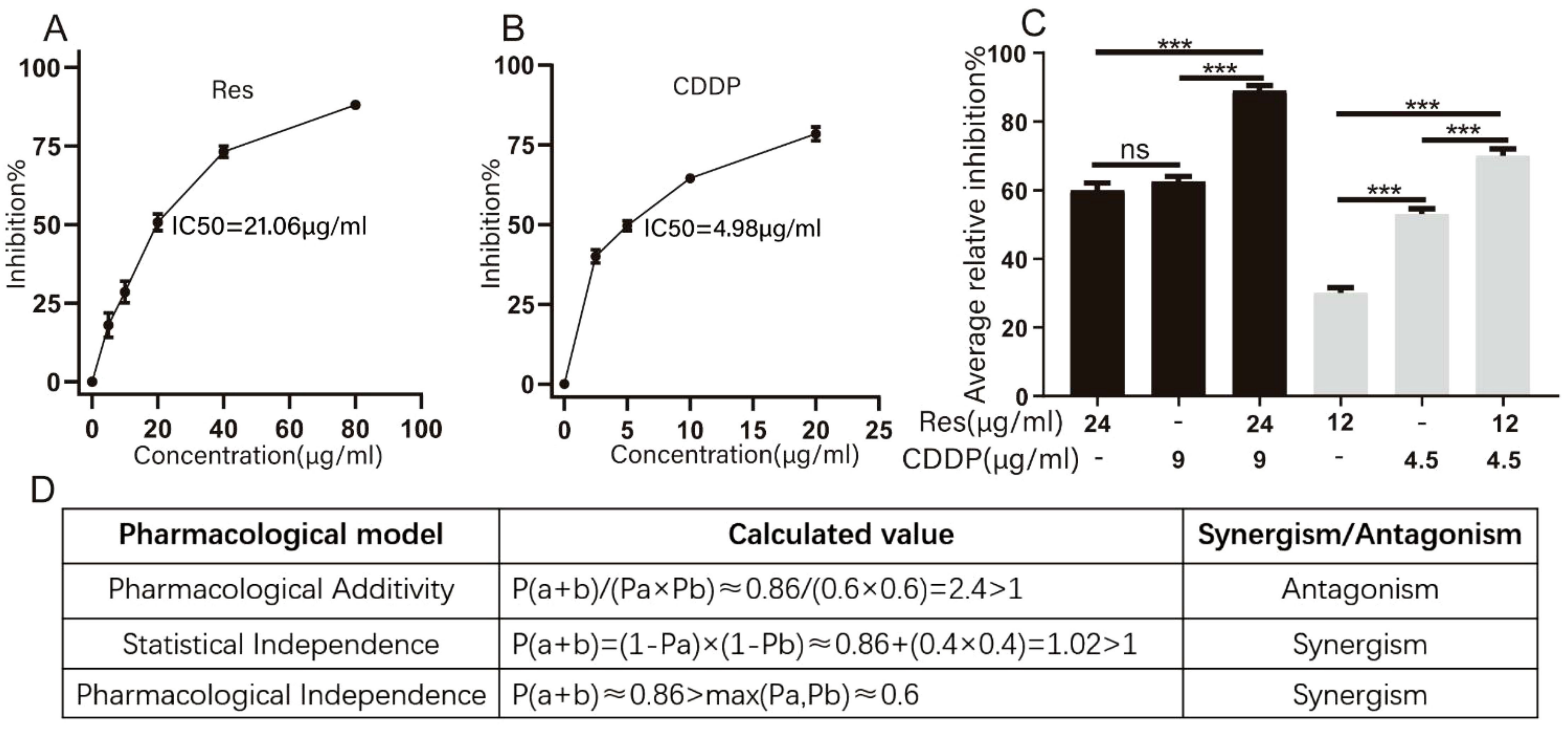
Resveratrol promotes cisplatin-induced HepG2 cell death. **(A)** Effects of different concentrations of resveratrol on survival of HepG2 cells. **(B)** Effects of different concentrations of cisplatin (CDDP) on survival of HepG2 cells (*n* ≥ 3; ^***^ *p* < 0.001). **(C)** Inhibition rate of HepG2 cells by different concentrations of resveratrol (24 μg/mL [105.15 μM], 12 μg/mL [52.57 μM]), cisplatin (9 μg/mL [30.00 μM], 4.5 μg/mL [15.00 μM]), and resveratrol combined with cisplatin (24 μg/mL [105.15 μM] + 9 μg/mL [30.00 μM]) (*n* ≥ 3; ^***^ *p* < 0.001). **(D)** Three models of evaluating drug interactions. P(a) represented the inhibition rate of resveratrol therapy. P(b) represented the inhibition rate of CDDP therapy. P(a + b) represented the inhibition rate of combination therapy.

First, the impact of different doses of Res or CDDP on HepG2 cells was tested. The results show that cell growth of HepG2 was inhibited in a dose-dependent manner by both Res and CDDP. We then calculated the IC_50_ values of Res and CDDP, which were 21.06 μg/mL (92.27 μM) and 4.98 μg/mL (16.60 μM), respectively (**Figures 1A, B**).

To investigate whether resveratrol treatment could promote cisplatin-induced HepG2 cell death, we measured the inhibition rate of HepG2 cells (**Figure 1C**) after treatment with different concentrations of resveratrol (24 μg/mL [105.15 μM] and 12 μg/mL [52.57 μM]) and cisplatin (9 μg/mL [30.00 μM] and 4.5 μg/mL [15.00 μM]). Drug interactions were evaluated based on Pharmacological Additivity model, Statistical Independence model, and Pharmacological Independence model (**Figure 1D**). The calculation results based on the Pharmacological Additivity models showed P(a + b)/(Pa × Pb) = 2.4 > 1, which indicated an antagonistic effect. Statistical Independence model showed P(a + b) = 0.88 and 1 − (1 − Pa) × (1 − Pb) = 0.84, P(a + b) + (1 − Pa) × (1 − Pb) < 1, so these two drugs were found to have a synergistic effect. The Independence result showed P(a + b) > max (Pa, Pb), which also suggested a synergistic effect. Generally, we thought that these two drugs exhibited synergy. Therefore, 24 μg/mL (105.15 μM) resveratrol and 9 μg/mL (30.00 μM) cisplatin were used for subsequent experiments.

### Resveratrol promotes cisplatin-induced HepG2 cell death through membrane-related pathways

**Figure 2:**
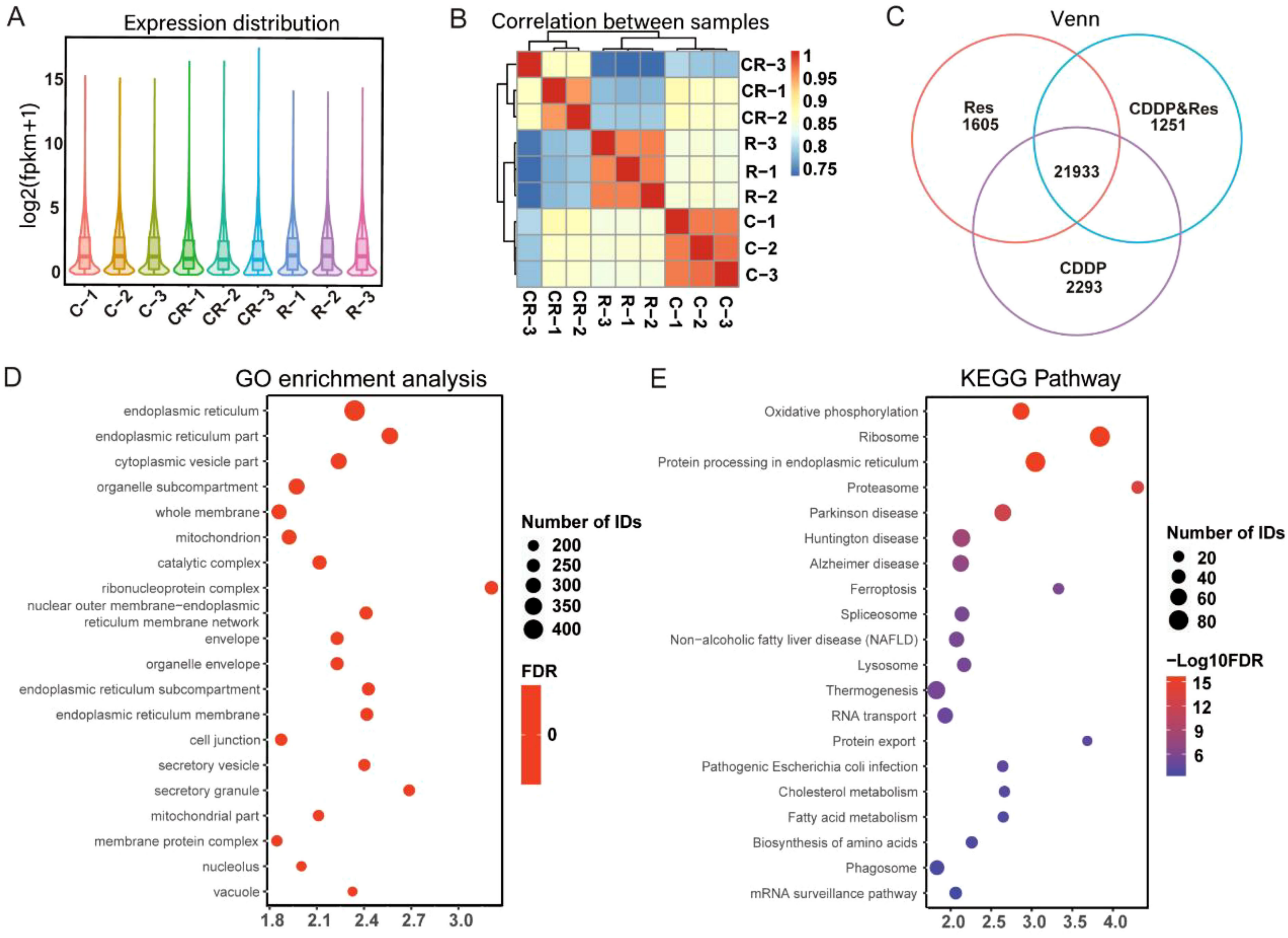
Resveratrol promotes cisplatin-induced HepG2 cell death through membrane-related pathways. **(A)** Expression distribution between samples. **(B)** Correlation analysis between samples. **(C)** Venn analysis of coexpressed genes. **(D)** Gene Ontology (GO) enrichment analysis of differentially expressed genes. **(E)** Kyoto Encyclopedia of Genes and Genomes (KEGG) pathway enrichment analysis of differentially expressed genes. The results shown in **(D, E)** were analyzed among three groups: R vs. C vs. CR.

To clarify how resveratrol treatment promoted cisplatin-induced HepG2 cell death, RNA-seq was performed on HepG2 cells after treatment with different groups of drugs (24 μg/mL [105.15 μM] resveratrol, 9 μg/mL [30.00 μM] cisplatin, and 12 μg/mL [52.57 μM] resveratrol + 4.5 μg/mL [15.00 μM] cisplatin). The results showed that the logarithm of FPKM in each sample is between 0.6 and 0.8 (**Figure 2A**), indicating that these genes were expressed at high levels. The correlation coefficients among the samples were all higher than 0.85 (**Figure 2B**), indicating a high degree of similarity in gene expression. The Venn diagram revealed 21,933 coexpressed genes and 1,605, 1,251, and 2,293 specifically expressed genes in the resveratrol group, CDDP group, and CDDP and resveratrol group, respectively (**Figure 2C**). GO analysis showed significant changes in cellular components related to the whole membrane (**Figure 2D**). KEGG pathway analysis showed that cholesterol metabolism and fatty acid metabolism rank high (**Figure 2E**). Together, these results suggest that resveratrol promotes cisplatin-induced HepG2 cell death through membrane-related pathways.

### Resveratrol changes the membrane component of HepG2 cells significantly

**Figure 3:**
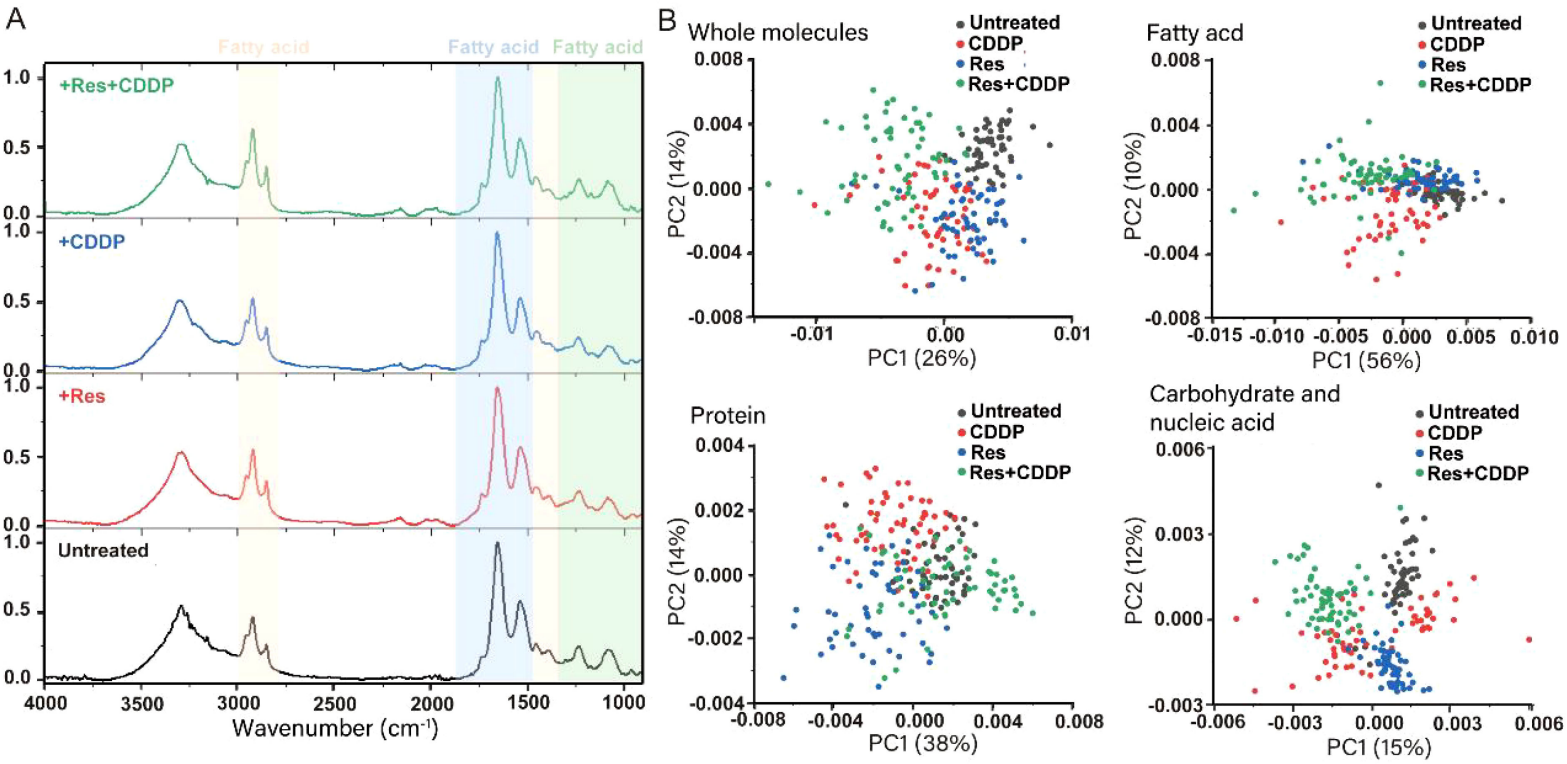
Resveratrol changes the component of the HepG2 cell membrane significantly. **(A)** Infrared average spectra of 200 HepG2 cells showing minor differences between the untreated group, 24 μg/mL resveratrol-treated group, 9 μg/mL cisplatin-treated group, and 24 μg/mL resveratrol combined with 9 μg/mL cisplatin-treated group. The orange part represents the fatty acid region of the cell (3,000–2,800 cm^−1^, 1,480–1,300 cm^−1^), the blue part represents the protein region (1,800–1,480 cm^−1^), and the green part represents the carbohydrate region (1,300–900 cm^−1^). **(B)** Principal component analysis (PCA) on second-derivative spectra of 200 HepG2 Cells. PCA analysis was performed based on the full spectrum region, fatty acid region, protein region, and carbohydrate region.

To test whether the membrane components of HepG2 cell were significantly changed by resveratrol, a single-cell infrared phenomics approach was applied to quantitatively evaluate cell heterogeneity within different drug treatment groups. HepG2 cells were treated with diverse drugs (24 μg/mL [105.15 μM] resveratrol, 9 μg/mL [30.00 μM] cisplatin, and 24 μg/mL [105.15 μM] resveratrol with 9 μg/mL [30.00 μM] cisplatin). The treated HepG2 were then dispersed into single cells, which were measured by infrared spectroscopy. A total of 200 single-cell spectra were collected, and **Figure 3A** demonstrates the average infrared spectra of these 200 HepG2 cells. After the preprocessing procedures described above, both the original absorbance spectra and the second-derivative spectra were calculated. PCA was carried out to reduce the dimension of the second-derivative spectra from the 200 cells. The principal components (PCs) were arranged according to their percentage of explained variance, from largest to smallest, and the first two PCs were chosen to construct the score plot (**Figure 3B**). PCA analysis was performed based on the full spectrum region, as well as the fatty acid, protein, and carbohydrate regions. In the full spectrum range, the cells from the untreated group and the combined resveratrol and cisplatin group clustered separately, while the cells of the resveratrol- and cisplatin-treated groups dispersed from each other, indicating that the biochemical components of the cells changed after drug treatment, with the combined treatment of resveratrol and cisplatin having a greater effect on the cells. The fatty acid components of several groups of cells were aggregated into different colonies, but the cells within each group were not completely dispersed, indicating that the fatty acid components of the cells were changed to a certain extent by drug treatment. Although the protein components of several groups of cells also formed different colonies, the dispersion between the single group of cells was low and the dispersion within each group of cells was large, indicating that the drug had little effect on the protein components of the cells, with a large difference in the protein components within each group. In the carbohydrate component region, the dispersion of cells in each group was large, with different clusters forming for each treatment, indicating that drug treatment caused obvious variation in the carbohydrate components of the cells. In summary, drug treatment had the greatest effect on carbohydrate components of cells, followed by fatty acids, with little effect on proteins.

### Resveratrol changed the morphology of the HepG2 cell membrane by attenuating the PLA2G2 expression

**Figure 4:**
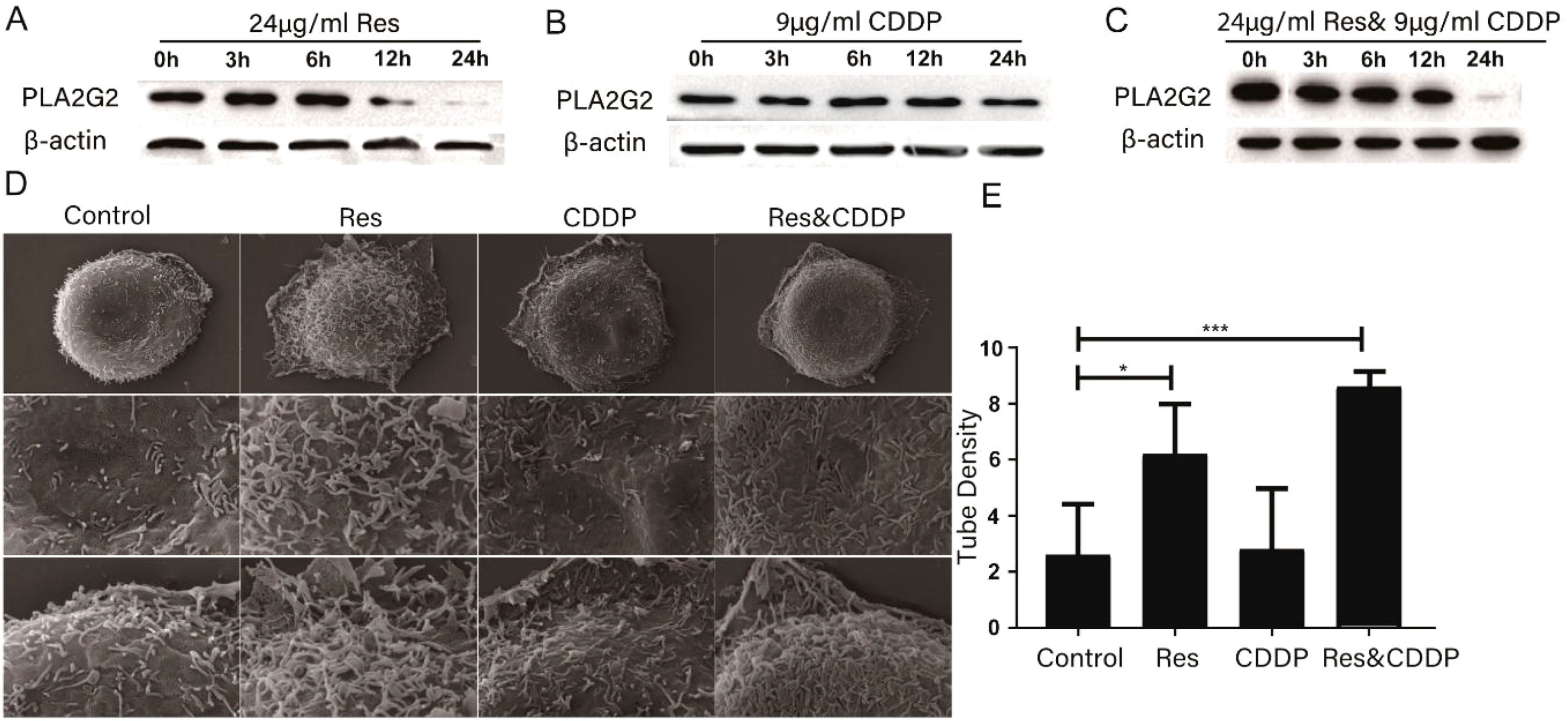
Resveratrol altered cell membrane morphology through PLA2G2. **(A)** Expression of PLA2G2 protein in HepG2 cells treated with resveratrol for 24 h with time. **(B)** Expression of PLA2G2 protein in HepG2 cells treated with CDDP for 24 h with time. **(C)** Expression of PLA2G2 protein in HepG2 cells treated with CDDP and resveratrol for 24 h with time. **(D)** Representative scanning electron microscope (SEM) images of HepG2 cells treated with different drugs for 24 h **(E)** Quantification showing tubule density affected by different drugs (*n* ≥ 8; ^*^ *p* < 0.05; ^***^ *p* < 0.001).

PLA2s are a type of phospholipase that can hydrolyze the sn-2 acyl bond of GPLs and disrupt cell membranes (21). Previous studies have shown that resveratrol can protect cell membranes from damage caused by PLA2 (21, 27–30). PLA2G2 has been studied in other diseases, such as dilated cardiomyopathy and obesity. We speculate that resveratrol may alter cell membranes by targeting the membrane protein PLA2s. Western blot was used to quantify PLA2G2 (one of the proteins in the PLA2 family) expression level after treatment with 24 μg/mL (105.15 μM) resveratrol and 9 μg/mL (30.00 μM) CDDP. The results showed that the expression level of PLA2G2 was attenuated after 3 h of treatment with resveratrol, while CDDP treatment had no effect on the expression level of PLA2G2 (**Figures 4A–C**).

We then used a scanning electron microscope to characterize HepG2 cells treated with 24 μg/mL resveratrol, 9 μg/mL (30.00 μM) cisplatin, and 24 μg/mL (105.15 μM) resveratrol combined with 9 μg/mL (30.00 μM) cisplatin for 24 h. Representative SEM images showed that the morphology of the HepG2 cell membrane undergwent significant changes. The membrane tubules in the drug-treated group were significantly fewer than those in the untreated group (**Figures 4D, E**), with the resveratrol-treated group showing the least membrane tubules among all groups. In conclusion, these results indicated that resveratrol changes the component of the HepG2 cell membrane significantly.

## Discussion

In 2020, approximately 906,000 people worldwide were diagnosed with cancer, with hepatocellular carcinoma being one of the most common cancers. Hepatocellular carcinoma is the third leading cause of cancer death globally, with a relative 5-year survival rate of approximately 18% (5). Chemotherapy is one of the most common methods of systemic therapies; however, advanced HCC is chemoresistant, which limits the available therapeutic options for patients (6). Furthermore, tumor heterogeneity has been identified as a crucial contributor to drug resistance, resulting in the failure of many therapeutic interventions (46). While cisplatin-based chemotherapy has been widely used in the treatment of HCC (7), resistance to cisplatin has emerged as a major obstacle. Consequently, there is a pressing clinical demand for the development of novel approaches to overcome cisplatin resistance and enhance sequential therapy for HCC. Resveratrol is a multitarget drug that has been shown to play a role in various functional features of cancer, such as sustained proliferative signaling (27, 47–49), resistance to programmed cell death (apoptosis) (50–53), and promotion of neovascularization (48, 49). There have been many reports on the combination of resveratrol and cisplatin for several cancers (16, 18, 19, 54). It can be seen that resveratrol is a very promising candidate for cancer combination therapy. Therefore, an in-depth exploration of the cancer treatment mechanisms of resveratrol is of great significance for its future application in combination therapies. In this study, we chose HepG2 cells to explore the anticancer mechanisms of resveratrol in combination with cisplatin.

The clinical correlation between the dosage and IC_50_ value of resveratrol is mainly reflected in its therapeutic effect on different diseases. Resveratrol, as a natural compound, exhibits various biological activities, including antitumor, anti-inflammatory, and hypoglycemic effects. The relationship between its dosage and IC_50_ value, that is, the inhibitory ability of resveratrol on cell growth at a specific concentration, is directly related to its therapeutic effects and possible side effects. In antitumor research, resveratrol has shown inhibitory effects on human colon cancer cells. Research has shown that the IC_50_ values of resveratrol on SW480 human colon cancer cells vary at different time points and exhibit a concentration-dependent apoptotic effect (55); however, some studies suggest that when the concentration exceeds a certain level, resveratrol can begin to cause toxicity to cells (56). This indicates that, within a certain dosage range, resveratrol can effectively inhibit the proliferation of tumor cells and induce their apoptosis.

Resveratrol has been reported to promote cisplatin-induced cancer cell death in many articles. Liu et al. proved that resveratrol has a synergistic effect of increasing the chemosensitivity of cisplatin on various hepatoma cells (43). Rahimifard’s research also indicated that these two drugs have a synergistic effect on the AGS and HEK293 cell lines (19). Similarly, Yang’s results confirmed this synergy in triple-negative breast cancer (57). The results mentioned above were all based on the Chou–Talalay model (Pharmacological Additivity model); however, in our study, we used the Pharmacological Additivity model (42), Statistical Independence model (44), and Pharmacological Independence model to evaluate drug efficacy. Our results, evaluated using the Pharmacological Additivity model, showed that the two drugs had antagonistic effects. This result differs from the case mentioned above, and the reason might be that we only evaluated two groups of drug concentrations. The results evaluated by the two other models showed that these two drugs showed a synergistic effect.

Our transcriptome sequencing results showed a high level of gene enrichment in the whole cell membrane pathway (**Figure 2D**), which made us speculate that resveratrol induced HepG2 cell death through a phase separation mechanism. Changes in the composition and content of the whole cell membrane may lead to phase separation pathways. For example, sphingolipids (SL) and cholesterol (Chol) are abundant in lipid rafts and form tight liquid-ordered (Lo) microregions. Regions with higher content of phosphatidylethanolamine (PE) have stronger fluidity and lower surface pressure, as the small polar heads of these lipids produce lower surface filling density and the formation of lipid-disordered (Ld) microregions (58). Phase separation is an important reason of cell apoptosis, which had been reported in numerous studies. For example, previous studies have shown that resveratrol induces apoptosis in multiple myeloma (MM) and T-cell leukemia cells through the co-aggregation of Fas/CD95 death receptors and lipid rafts (59). However, another study showed that *N*-(3-oxododecanoyl) homoserine lactone was incorporated into the mammalian plasma membrane and induced dissolution of eukaryotic lipid domains, which expelled tumor necrosis factor receptor 1 into the disordered lipid phase for its spontaneous trimerization (60). Therefore, we speculate that the combination of resveratrol and cisplatin induces phase separation, leading to the activation of apoptotic receptors, which further induces cell apoptosis.

We proposed a novel strategy for quantitatively evaluating cellular heterogeneity by statistical analysis of single-cell infrared spectra obtained from synchrotron FTIR microspectroscopy (61, 62). These infrared spectra contain various chemical component information of the cells, such as fatty acids, proteins, and nucleic acids. Our previous articles indicated that this established infrared phenomics approach can be used to find clues in the correlation between the drug-induced phenotypic changes and its potential target (63). Based on this strategy, the cancer cell HepG2 glycocalyx was first identified as a potential target of protopanaxadiol, an herbal medicine. These findings provide a powerful tool for accurately evaluating cell stress responses and largely expanding the phenotypic screening toolkit for drug discovery (63). In this study, we used single-cell infrared phenotype omics methods to characterize the heterogeneity of HepG2 cells. The results showed that resveratrol treatment had a significant impact on the fatty acids and sugar layer of HepG2 cells (**Figure 3B**). Additionally, scanning electron microscopy confirmed the changes of the cell membrane (**Figures 4C, D**). Overall, these experimental results further confirm that resveratrol may induce HepG2 cell death through a phase separation mechanism. Huang et al. found that cisplatin enhanced FAS-mediated apoptosis through lipid rafts (64). Similarly, Lacour et al. reported that cisplatin activates aSMase and induces ceramide production, which triggers the redistribution of CD95 into the plasma membrane rafts (65).

Phospholipase A2 enzymes hydrolyze the sn-2 acyl bond of glycerophospholipids, releasing lysophospholipids and free fatty acids, mainly polyunsaturated fatty acids (66). Previous studies have shown that resveratrol can protect cell membranes from PLA2 damage. Fei et al. conducted molecular dynamics simulations on the bilayer of dipalmitoyl phosphatidylcholine containing resveratrol and found that resveratrol can protect the sn-1 and sn-2 ester bonds of DPPC and DSPC from being cleaved by phospholipase A1 and phospholipase A2 (67, 68). Shukla et al. used fluorescence spectroscopy and surface plasmon resonance techniques to show that PLA2 interacts with resveratrol with high binding affinities (27). Xu et al. found that resveratrol inhibited lipoprotein-associated phospholipase A2 in rabbits fed a high-fat diet (28). In this study, we confirmed that resveratrol attenuated the PLA2G2 expression in HepG2; however, CDDP had no significant effect on PLA2G2 (**Figure 4A**). We have also provided relevant reviews on the correlation between PLA2G2 and cancer. These articles all show that the PLA2G2 protein is upregulated in cancer expression (69–71). We have also found two research articles on PLA2G2 and other diseases (72, 73). The articles mentioned above are listed in **Table 1**. It can be seen that PLA2G2 activity changes significantly in some disease states, and PLA2G2 plays a key role in disease mechanisms.

**Table 1:** The association between PLA2 and disease.

Disease	Association	Reference
Dilated cardiomyopathy	PLA2G2 decreased significantly in patients with DCM compared with nonfailing donors	Yaohan Tang et al. (74)
Obesity	HFD feeding significantly decreased the expression of regenerating Islet-derived 3-gamma (Reg3g) and phospholipase A2 group-II (PLA2g2) in the jejunum	Everard et al. (73)
Ovarian carcinoma	sPLA2-IIA was found to be upregulated prior to chemotherapy but significantly downregulated postchemotherapy	Bristow et al. (75)
Prostate cancer	Secreted PLA2-IIA was increased in prostate cancer tissues	Jiang et al. (76)
Prostate tumor	sPLA2-IIa expression was increased dramatically in the androgen-independent CWR-22R and LNAI CaP cells versus the androgen-dependent CWR-22 and LNCaP cells	Graff et al. (77)

In conclusion, we provided further insights into the antiliver cancer effects of resveratrol. We demonstrated that Res promoted cisplatin-induced HepG2 cell death by attenuating PLA2G2 expression and membrane-related pathways. Moreover, cisplatin combined with resveratrol can exert a synergistic therapeutic effect on HepG2 cells. Thus, these findings propose a potential strategy for the drug treatment of liver cancer.

## Data Availability

The raw data supporting the conclusions of this article will be made available by the authors, without undue reservation.
